# Birth experience in newborn infants is associated with changes in nociceptive sensitivity

**DOI:** 10.1038/s41598-019-40650-2

**Published:** 2019-03-11

**Authors:** Severin Kasser, Caroline Hartley, Hanna Rickenbacher, Noemi Klarer, Antoinette Depoorter, Alexandre N. Datta, Maria M. Cobo, Sezgi Goksan, Amy Hoskin, Walter Magerl, Evelyn A. Huhn, Gabrielle Green, Rebeccah Slater, Sven Wellmann

**Affiliations:** 1 Division of Neonatology, University of Basel Children’s Hospital (UKBB), Basel, Switzerland; 2 Department of Paediatrics, University of Oxford, Oxford, United Kingdom; 3 Division of Neuropediatrics & Developmental Medicine, University of Basel Children’s Hospital (UKBB), Basel, Switzerland; 4 Department of Neurophysiology, Center of Biomedicine and Medical Technology Mannheim (CBTM), Medical Faculty Mannheim, University of Heidelberg, Mannheim, Germany; 5 Department of Obstetrics and Gynaecology, University Hospital Basel, Basel, Switzerland; 6 Department of Neonatology, University Children’s Hospital Regensburg (KUNO), University of Regensburg, Regensburg, Germany

## Abstract

Vaginal birth prepares the fetus for postnatal life. It confers respiratory, cardiovascular and homeostatic advantages to the newborn infant compared with elective cesarean section, and is reported to provide neonatal analgesia. We hypothesize that infants born by vaginal delivery will show lower noxious-evoked brain activity a few hours after birth compared to those born by elective cesarean section. In the first few hours of neonatal life, we record electrophysiological measures of noxious-evoked brain activity following the application of a mildly noxious experimental stimulus in 41 infants born by either vaginal delivery or by elective cesarean section. We demonstrate that noxious-evoked brain activity is related to the mode of delivery and significantly lower in infants born by vaginal delivery compared with those born by elective cesarean section. Furthermore, we found that the magnitude of noxious-evoked brain activity is inversely correlated with fetal copeptin production, a surrogate marker of vasopressin, and dependent on the experience of birth-related distress. This suggests that nociceptive sensitivity in the first few hours of postnatal life is influenced by birth experience and endogenous hormonal production.

## Introduction

Natural birth is an unparalleled life stressor. Labor induces the production of fetal stress hormones including catecholamines, cortisol, and vasopressin^1^. These hormones are thought to help the fetus adapt to the extra-uterine environment^1^, and are implicated in providing analgesia during birth and immediate postnatal life^2,3^. For example, it has been reported that vaginally-born infants manifest dampened behavioral and physiological pain-related responses, compared with infants born by cesarean section, following intramuscular injections performed shortly after birth^2^.

Recently electrophysiological measures have been developed which enable the objective quantification of noxious-evoked activity in the infant brain^4,5^. These measures are validated for both clinical and experimental noxious procedures and are sensitive to analgesic modulation^6^. Cortical activation is considered to be a fundamental requirement for an experience to be interpreted as painful^7^, therefore, inferences about infant pain perception that are based on brain-derived measures may have advantages over traditional pain assessment techniques as they are not reliant on the measurement of motor and autonomic responses^8^. Noxious-evoked brain activity can be measured shortly after birth and can be used to assess whether individual differences in infant pain sensitivity relates to birth experience.

The production of fetal vasopressin has the potential to confer endogenous analgesia to the newborn infant. In both rodents and adult man, vasopressin is an effective central and peripheral acting analgesic^9,10^. Although the direct measurement of vasopressin concentration is difficult, copeptin is an excellent surrogate marker. It is derived from the same precursor molecule as vasopressin, produced in an equimolar ratio and is a more stable peptide^11^. The concentration of copeptin, dramatically increases during vaginal birth, and its concentration in umbilical cord blood is approximately 100 times higher in healthy infants born by vaginal delivery compared to those delivered by elective cesarean section^12^. This leads to the possibility that birth-related stress drives an increase in fetal vasopressin production which leads to endogenous analgesia being conferred to the newborn infant.

Here we hypothesize that infants born by elective cesarean section will show heightened noxious-evoked brain activity a few hours after birth compared to those born by vaginal delivery in response to a mild noxious experimental stimulus. We investigate whether the magnitude of the noxious-evoked brain activity is related to mode of delivery; and, as secondary outcomes, whether the magnitude of noxious-evoked brain activity is related to fetal vasopressin production, and dependent on the occurrence of birth-related fetal distress.

## Results

### Birth experience influences infant nociceptive sensitivity

**Figure 1:**
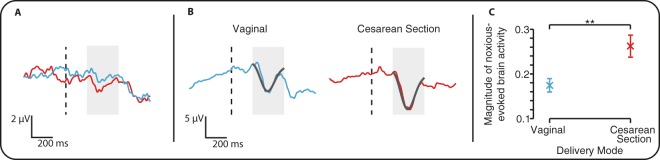
Noxious-evoked brain activity is higher in infants born by elective cesarean section. (**A**) Average EEG activity recorded in response to the experimental noxious stimulation in the infants born by vaginal delivery (blue) and elective cesarean section (red). Dashed black line indicates the point of stimulation and the shaded area indicates the time window of the evoked response. (**B**) Group averages after the data has been latency jittered to account for individual variation in the response latency within the time window of interest 200–500 ms after the stimulus (shaded region). The template of the nociceptive response, which has been scaled to fit the evoked activity, is shown overlaid in grey. (**C**) The magnitude of the noxious-evoked brain activity (characterized by the scaled template – see Methods) for the two groups, shown adjusted for gestational age and sex. (Error bars: mean ± standard error of the mean, **p < 0.01, multiple linear regression, n = 22 infants born by vaginal delivery and 19 infants born by elective cesarean section).

Evoked brain activity was recorded using EEG in response to experimental noxious stimuli applied to the hand in 41 term infants 4.6 hours (±0.8, mean ± standard deviation) after birth (Fig. 1). Noxious-evoked brain activity was significantly higher in infants born by elective cesarean section (n = 19) compared with infants born by vaginal delivery (n = 22, Fig. 1C, p = 0.008, regression coefficient β = 0.088, 95% CI: [0.025, 0.15]). There was no significant effect of gestational age or sex (p = 0.50 and 0.96 respectively), which were included in the statistical model.

### Metabolic stress is increased in vaginally-born infants

**Figure 2:**
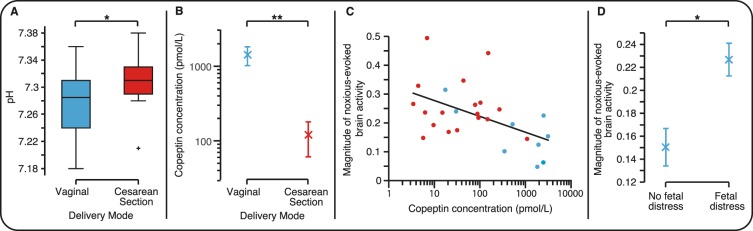
Nociceptive sensitivity is dependent on copeptin levels and fetal distress. (**A**) Umbilical artery pH in the infants born by vaginal delivery (n = 18) compared with those born by elective cesarean section (n = 18). (**B**) Copeptin concentration in the infants born by vaginal delivery (n = 9) compared with those born by elective cesarean section (n = 18). (**C**) Copeptin levels compared with the magnitude of the noxious-evoked brain activity (adjusted for gestational age) in the infants born by vaginal delivery (blue) and elective cesarean section (red). (**D**) The magnitude of the noxious-evoked brain activity (adjusted for time since rupture of membranes, length of second stage of labor, regional anesthesia during labor, sex and gestational age, multiple linear regression) in the infants who experienced distress during labor and delivery (n = 8) compared with those who did not (n = 14) in the infants born by vaginal delivery. (Note: data in Figure 2A, B & C is only reported where adequate blood samples were obtained – see Methods. Error bars: mean ± standard error of the mean, **p < 0.01, *p < 0.05).

Vaginally-born infants experience increased metabolic stress compared with infants born by elective caesarean section^13^. Umbilical artery pH, which is a marker of metabolic stress, has previously been shown to be lower in infants born by vaginal delivery, and is considered to be pathological when less than 7^14^. For all infants in this study the umbilical artery pH was in the clinically normal range (range 7.18–7.38), however, vaginally-born infants had significantly lower umbilical artery pH compared to those born by elective cesarean section (Fig. 2A, vaginally delivered infants pH = 7.29 (0.07), median (interquartile range), elective cesarean section pH = 7.31 (0.04), p = 0.043, Mann-Whitney-U test, n = 18 infants in each group - where adequate blood samples for analysis were obtained).

### The magnitude of noxious-evoked brain activity in infants is related to fetal copeptin concentration

As a secondary outcome of the study, we next investigated whether the noxious-evoked brain activity was related to copeptin concentration in a subsample of the infants where adequate cord blood samples were obtained. Consistent with previous research, infants born by vaginal delivery had significantly higher copeptin concentration in the cord blood compared with infants born by elective cesarean section (Fig. 2B, p < 0.001, t-test comparing the logarithm of copeptin concentration between the two groups, n = 9 vaginally delivered infants, 18 infants born by elective cesarean section - where adequate blood samples for analysis were obtained). Increased concentration of cord blood copeptin was also significantly correlated with a decrease in the magnitude of noxious-evoked brain activity (Fig. 2C, p = 0.019, correlation coefficient r = −0.46, β = −0.055, 95% CI: [−0.10, −0.01], logarithm of copeptin concentration compared with the magnitude of the noxious-evoked brain activity, adjusting for gestational age [p = 0.058], n = 27). As cord blood copeptin concentration is also dependent on mode of delivery, the regression will be influenced by delivery mode. However, even when only considering infants born by vaginal delivery, where a substantially greater range of copeptin values were observed (copeptin concentration: vaginally-born infants: 17–3138 pmol/L; caesarean-born infants: 3–1087 pmol/L), a significant negative correlation between copeptin concentration and the magnitude of the noxious-evoked brain activity was still observed (after adjusting for age, p = 0.035, β = −0.072, CI: [−0.14, −0.007], n = 9).

### Fetal distress during vaginal delivery increases noxious-evoked brain activity

In this study, none of the infants born by vaginal delivery required an assisted delivery (which was a predefined exclusion criterion). However, even when birth is straightforward, the experience can differ substantially between infants leading to differing levels of birth-related stress. As a secondary outcome of the study, we therefore considered whether time since rupture of membranes, length of the second stage of labor, regional anesthesia during labor, and the occurrence of fetal distress during vaginal delivery influenced nociceptive sensitivity after birth. We observed that the magnitude of the noxious-evoked brain activity was significantly higher in infants who had experienced fetal distress (identified by the presence of meconium stained amniotic fluid or non-reassuring fetal heart rates; Fig. 2D, p = 0.035, β = 0.076, CI: [0.006, 0.15]; infants with fetal distress n = 8; infants without fetal distress n = 14). Time since rupture of membranes (mean ± standard deviation: 386 ± 554 minutes), length of second stage of labor (73 ± 58 minutes), maternal regional anesthesia during labor (n = 11), sex and gestational age did not significantly influence the magnitude of the noxious-evoked activity recorded in the infants after birth (p > 0.05). In the sample of infants where adequate blood samples were obtained for copeptin analysis (4 infants with fetal distress and 5 without fetal distress), despite the low numbers it is of interest that 3 of the 4 infants with fetal distress had copeptin values higher than the infants without fetal distress (infants without fetal distress: range of copeptin concentrations, 17.5–1930.0 pmol/L, infants with fetal distress: copeptin values, 346.9, 2501.0, 2468.0, and 3138.0 pmol/L).

## Discussion

The process of vaginal birth prepares the fetus for the extra-uterine environment; it confers respiratory, cardiovascular and homeostatic advantages to the newborn infant^15,16^, and is considered to play a critical role in breastfeeding and infant-mother bonding^17,18^. Hormones produced during labor and birth are thought to provide endogenous analgesia in the early postnatal period^2,3^. Here we show that in early postnatal life, infants born by vaginal delivery have a reduction in the magnitude of their noxious-evoked brain activity compared to infants born by elective cesarean section, and that this effect is related to birth-stress and the production of fetal copeptin, a surrogate marker of vasopressin.

In animals, endogenous and exogenous increases in vasopressin are thought to produce pain inhibition^10^ and vasopressin-1a receptors located in dorsal root ganglia have been identified as analgesic targets^19^. Consistent with these observations, an analgesic effect has been observed in adult man when the synthetic vasopressin analog desmopressin is administered intranasally^9^. In the fetus, vasopressin concentrations rise sharply during vaginal birth to levels that are unparalleled by any other life experience^12^. Thus, vasopressin is a strong candidate for mediating the dampened nociceptive response that we have observed in the newborn infant shortly after vaginal delivery. However, the concentration of other hormones, such as oxytocin, cortisol and catecholamines^20,21^ are also dramatically altered by birth and may directly contribute to the reduction in noxious-evoked brain activity observed in this study. While the concentration of fetal oxytocin is likely to influence our results, in contrast to rodents, there is little transport of maternal oxytocin across the human placenta, and fetal oxytocin does not increase during labor^22^. Nevertheless, the conclusions we can draw are limited because we did not measure the concentration of other hormones or other factors that may influence nociceptive sensitivity after birth. The correlation we observe between copeptin concentration and the magnitude of noxious-evoked brain activity does not imply that increased vasopressin concentration is solely causing the reduction in noxious-evoked activity, but rather that it is one factor that may contribute to the differences in nociceptive activity observed between the group.

Infants born by vaginal delivery experience greater metabolic stress than infants born by elective cesarean section^23^. They have significantly lower umbilical cord pH and significantly greater copeptin concentrations than infants born by vaginal delivery^12^. Indeed, even small numbers of uterine contractions are sufficient to increase copeptin concentrations in the fetus^24^. These observations are consistent with animal studies that demonstrate a dose-dependent link between uterine contractions, minor hypoxia and subsequent vasopressin release^25,26^. This strongly indicates that normal vaginal delivery increases metabolic stress and causes an increase in the production of fetal vasopressin^27^. Although copeptin concentration was not recorded in all infants in our study (adequate blood samples are more difficult to obtain in vaginally delivered infants), we show that increased fetal copeptin inversely correlates with the magnitude of noxious-evoked brain activity recorded in the newborn infant a few hours after birth.

Some of the vaginally-born infants in our study experienced fetal distress, which was defined as the presence of meconium stained amniotic fluid or the recording of non-reassuring fetal heart rates. Greater noxious-evoked brain activity was recorded in these infants compared with the vaginally-born infants who did not experience fetal distress. This is consistent with the observation that infants who require assisted vaginal delivery, who are more likely to have experienced fetal distress, display exaggerated pain-related behaviors on the third day of life compared to infants born by unassisted vaginal delivery or elective cesarean section^28^.

Given the higher levels of copeptin recorded in the vaginally-born infants, we suggest that normal levels of birth-related stress associated with uncomplicated vaginal delivery leads to a proportional increase in the production of fetal vasopressin, and increased endogenous analgesia. However, for infants who experience fetal distress, which is likely to be associated with a substantial increase in metabolic stress, the analgesic benefits conferred to the infant via the production of fetal vasopressin is less efficacious. This is consistent with adult data that shows that the efficacy of vasopressin analgesia is reduced in more highly stressed males^9^.

When abnormally high levels of fetal stress are experienced by an infant, for example during assisted vaginal delivery or birth asphyxia, there is an increase in copeptin concentration as compared with normal vaginal delivery^12,29^. Interestingly, in rats intravenous injection of vasopressin strongly reduces noxious-evoked action potential firing, however, administration of extremely high concentrations of vasopressin leads to the opposite facilitatory effect, where an increase in action potential firing is observed^10^. It is also likely that fetal distress is correlated with other birth factors such as the length of labor and birth parity, however, this study was not powered to disentangle these effects.

Previous studies in infants have demonstrated sex-dependent differences in pain sensitivity, with male infants crying sooner and longer in response to a heel-lance performed in the first days after birth^30^ and noxious-evoked increases in hemoglobin concentration in the somatosensory cortex more pronounced in male infants^31^. Given the huge quantities of fetal vasopressin produced during birth, and the fact that copeptin expression is higher in males^32^, it is plausible that vasopressin may confer sex-dependent effects that influence nociceptive sensitivity in the newborn infant. Whilst we did not find sex-dependent differences in the noxious-evoked responses recorded in this study, this was not our primary outcome measure and so we were likely underpowered to observe such effects.

A limitation of this study is that we do not know whether the effects observed here are specific to nociceptive processing. It is possible that brain activity evoked by other non-noxious tactile stimulation may also be dampened following vaginal delivery; this requires further investigation. Furthermore, mode of delivery could impact the background EEG activity, and this could potentially influence the magnitude of evoked activity characterized here. For example, while none of the infants included in this study showed visible signs of head swelling or had a caput succedaneum, we cannot rule out the possibility that vaginal delivery could alter background EEG activity in these infants compared with those born by caesarean section. Nevertheless, a previous study that compared amplitude-integrated EEG activity in infants born vaginally or via caesarean section did not identify differences in brain activity, unless a caput succedaneum was present^33^.

In summary, we have used a sensitive and objective measure of noxious-evoked brain activity, and have demonstrated that at approximately 5 hours after birth, infants born by elective cesarean section have greater noxious-evoked brain activity to acute experimental noxious stimuli compared with infants born by unassisted vaginal delivery. A surge of fetal stress hormones during vaginal delivery may play an important role in priming the fetus for postnatal adaptation by providing endogenous analgesia during delivery and in the first few hours of postnatal life.

## Methods

This prospective study was conducted at the University Hospital Basel, Switzerland, with enrolment from January to May 2016. The Competent Ethics Committee of Northwestern Switzerland approved the study (EKNZ 2015-079), and written informed consent was obtained from the parents. All experiments were performed in accordance with relevant guidelines and regulations. Infants were included if they were from a singleton pregnancy and delivered at 37 weeks’ gestation or above, either by elective cesarean section without preceding contractions or rupture of the membranes, or by spontaneous vaginal delivery without any instrumental support. Exclusion criteria were infants with chromosomal aberrations, malformations, birth defects, admission to neonatal intensive care, use of instrumental support during vaginal delivery, the commencement of labor prior to planned cesarean section, and infants born to mothers where substance abuse, infection, hypertension, preeclampsia or diabetes type I or II occurred during pregnancy.

### Infant Demographic Characteristics

**Figure 3:**
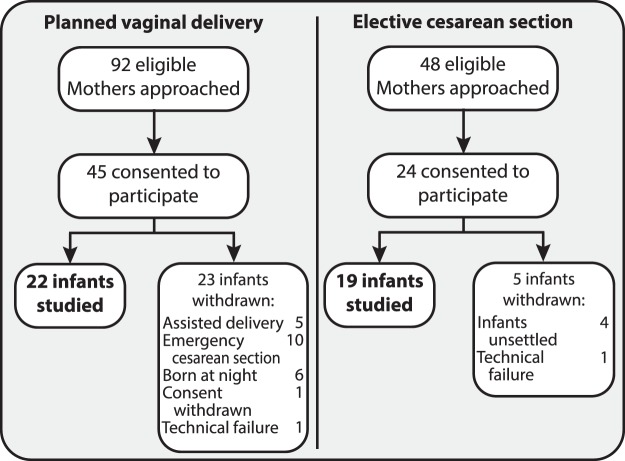
Study flowchart.

Parents who expected to deliver their infants by vaginal delivery (n = 92) or elective cesarean section (n = 48) were approached about the study, of which 22 infants born by vaginal delivery and 19 born by elective cesarean section were included (Fig. 3). Infants were delivered by elective cesarean section based on clinical judgment or parental request. Infant demographics are shown in Table 1. There were no significant differences between the two groups in factors such as birth weight, Apgar scores or postnatal age. However, maternal regional anesthesia differed between the two groups; all women who had a cesarean section received spinal anesthesia by single-shot technique using 10 mg 0.5% hyperbaric bupivacaine and 10 mcg fentanyl, 11 of 22 women who had a vaginal delivery received an epidural analgesia using a ready-mix of bupivacaine 0.1% and fentanyl 2 mcg per ml. None of the women received systemic opioids before delivery. All women who had a cesarean section received 100 mcg morphine via spinal catheter after delivery, and the women who had a vaginal delivery without epidural analgesia received non-opioid pain relief during labor. None of the infants had a caput succedaneum. The infants born by elective cesarean section were significantly younger (see Table 1). As the magnitude of noxious-evoked brain activity increases with gestational age^34^, this factor was included in all of the statistical models.

**Table 1:** Infant demographics.

Variable	Cesarean delivery (n = 19)	Vaginal delivery (n = 22)	p
Maternal age (years)	33 (22–42)	34 (24–40)	n.s.
Multiparous	15 (79)	15 (68)	n.s.
Maternal regional anesthesia	19 (100)	11 (50)	0.001
Gestational age (days)	273 (266–285)	279 (262–289)	0.001
Birth weight (g)	3270 (2810–4300)	3528 (2820–4350)	n.s.
Birth length (cm)	48 (47–51)	50 (48–52)	n.s.
Head circumference (cm)	35 (32–38)	35 (32–37)	n.s.
Neonatal sex, female	9 (47)	11 (50)	n.s.
5-min Apgar score	10 (8–10)	10 (8–10)	n.s.
Time elapsed from birth to EEG recording (minutes)	284 (219–351)	265 (138–345)	n.s.

Data are median (range) or n (%). Mann-Whitney, Chi-squared or Fisher exact tests were used as appropriate to compare groups at a 95% significance level. n.s. = not significant. Regional anesthesia was a spinal anesthetic in the case of cesarean delivery or an epidural in the case of vaginal delivery.

### Study design

#### EEG recordings

EEG was recorded with the Brainvision standard V-Amp amplifier (Brain Vision LLC, Morrisville, USA) with a sampling rate of 2000 Hz. According to the international 10–20 system, EEG activity was recorded at the Cz electrode and referenced to A1, with the ground electrode on the forehead. EEG conductive paste was used to optimize contact with the scalp and to minimize impedance.

#### Experimental noxious stimulation

Experimental noxious stimuli were applied with a weighted mechanical stimulus (Pinprick, MRC Systems, Heidelberg, Germany) calibrated to apply a force of 32 mN. The point of stimulation was directly linked to the EEG recordings at the time of acquisition using a contact trigger device (MRC Systems, Heidelberg, Germany). In infants these stimuli evoke nociceptive brain activity without causing behavioral distress^4^. Application time for all stimuli was approximately 1 second with an inter-stimulus interval of at least 3 seconds to avoid pain summation^35^. 50 stimuli were applied to the dorsal surface of the infant’s right hand. Recording and stimulation techniques are described in detail by Klarer *et al*.^36^.

#### Copeptin and pH

Cord blood samples were drawn immediately after birth, by puncture of the umbilical artery, for pH determination and for centrifugation of serum. The concentration of copeptin in arterial cord blood was measured in all sufficient blood samples that were collected after pH analysis. Samples were first frozen to a temperature of −28 °C and then copeptin measurement was done in a single batch using the BRAHMS Kryptor Compact immunoanalyzer (Thermo Scientific Brahms GmbH, Hennigsdorf, Germany). The lower detection limit was 0.9 pmol/L, and the functional assay sensitivity (20% interassay coefficient of variance [CV]) was 2 pmol/L. The inter-assay precision was <7% CV at 5 pmol/L and <4% CV at 100 pmol/L.

### Data analysis

#### EEG analysis

The EEG signals were imported into MATLAB using EEGLAB (Swartz Center for Computational Neuroscience, University of California San Diego) and filtered with a high pass filter at 1 Hz and a low pass filter at 30 Hz. Each stimulus response was extracted in 1500 ms epochs, with 500 ms before the stimulus, and baseline corrected to the pre-stimulus mean. EEG epochs with artefacts, such as gross movement artefacts, were rejected from the analysis.

The data was Woody filtered (latency jittered), with a maximum jitter of ±50 ms in the time window 0–600 ms after the stimulus, by maximizing the cross-correlation of the individual trials with the group average. This was performed for each group (i.e. vaginally delivered or elective cesarean section) separately. A template of noxious-evoked brain activity defined in an independent data set^6^ was then projected onto each individual trial as previously described^4,6,34,37^. The template represents the waveform of the noxious-evoked brain activity within the time window of interest and by projecting the template onto the data the magnitude of this waveform within the data (the scaling factor of the template) can be calculated^6^. Using this approach, the magnitude of noxious-evoked brain activity in each individual trial was calculated in the time window 200–500 ms after the stimulus (see Section *Identifying the latency of the noxious-evoked response*).

As Woody filtering will naturally increase any deviations within the data and therefore can erroneously suggest an evoked response is present, the results were compared with background activity. The data was Woody filtered, with a maximum jitter of ±50 ms in the time window −400 to 0 ms before the stimuli, by maximizing the cross-correlation of the individual trials with the group average, and the template was projected onto the data in the time window −350 to −50 ms before the stimulus. The magnitude of the noxious-evoked brain activity was significantly higher following the stimulus compared with background data (p < 0.001, paired t-test).

#### Identifying the latency of the noxious-evoked response

Noxious-evoked brain activity has previously been well characterized in response to stimuli applied to the foot^5,6,37^. However, there is likely to be a latency difference to the response when stimuli are applied to the hand. In an independent sample of 14 infants we therefore first characterized the response to experimental noxious stimuli applied to the hand, determining the latency to the response and confirming that the template of noxious-evoked brain activity was suitable for identifying the response in term infants.

For this independent study, infants were recruited from the Maternity Unit at the John Radcliffe Hospital, Oxford University Hospitals NHS Foundation Trust, UK. Infants were between one and four day’s postnatal age at time of study and aged 37 to 42 weeks’ gestation at birth. Infants were eligible for inclusion in the study if they were clinically stable, had no requirements for respiratory support and were not receiving analgesics. Infants born by any mode of delivery were included. Ethical approval (National Research Ethics Service, REC reference: 12/SC/0447) and informed written parental consent was obtained.

EEG activity was acquired with the SynAmps RT 64-channel headbox and amplifiers (Compumedics Neuroscan), with a bandwidth from DC-400 Hz and a sampling rate of 2000 Hz. CURRYscan7 neuroimaging suite (Compumedics Neuroscan) was used to record the activity. EEG recording electrodes (Ambu Neuroline disposable Ag/AgCl cup electrodes) were positioned at Cz, CPz, C3, CP3, C4, CP4, FCz, Oz, T3 and T4, according to the modified international 10–20 electrode placement system. Reference and ground electrodes were placed at Fz and on the forehead respectively. Impedance was minimised by gently rubbing the skin with EEG preparation gel (Nuprep gel, D.O. Weaver and Co.) and conductance paste (Elefix EEG paste, Nihon Kohden) was used to optimise skin contact.

Experimental noxious stimuli (PinPrick, calibrated to a force of 128 mN) were applied to the dorsum of the infant’s hand and the heel of the foot in trains of approximately 10. In 7 studies, the experimental noxious stimuli were time-locked to the EEG recordings using a high-speed camera (Firefly MV, Point Grey Research Inc.)^4^. In the other 7 studies, the stimuli were time-locked to the EEG recordings using a contact trigger device (MRC systems). The order (hand or foot) and the side were randomly selected (right hand side in 5 infants, left hand side in 9 infants). Before the stimuli, background activity was recorded where the infant’s foot or hand was gently held but no stimuli were applied.

The EEG signal at the Cz electrode was filtered 0.5–70 Hz with a notch filter at 50 Hz. 1500 ms epochs were extracted with 500 ms before the stimulus and traces were baseline corrected to the pre-stimulus mean. Individual epochs were rejected if they were contaminated by artefact. Two infants’ hand responses and another 2 infants’ foot responses were rejected completely. Individual EEG trials (in response to stimulation on the hand, foot, and in background activity separately) were Woody filtered in the interval 0–700 ms after the stimulus onset with a maximum shift of ±50 ms to the data average. Trials were then averaged across individual infants, and the infants’ responses to stimulation on the foot and stimulation on the hand compared separately with background activity using non-parametric cluster analysis^38^. Clusters of significant activity were defined as consecutive time points where significant differences in the response to the experimental noxious stimulus and background activity were identified, and the cluster-based test statistic was calculated from 1000 random permutations of the data^6,38^.

Consistent with previous studies, applying experimental noxious stimuli to the foot evoked a significant response in the time window 459–664 ms after the stimulus (p = 0.01, cluster-based test statistic)^6^. In contrast, the non-parametric cluster analysis identified an earlier significant evoked response from 289–438 ms following stimulation of the hand (p = 0.01, Supplementary Fig. 1). To obtain a representative waveform of the entire evoked response, Principal Component Analysis was conducted in the time window 200–500 ms after the stimulus, comparing the responses to stimulation on the hand and background activity. The weights of the first principal component (PC) were not significantly different between the background and the stimulus response (p = 0.10, t-test), indicating that the PC was not related to the stimulus response. In contrast, the weights of the second PC were significantly higher following the noxious stimulus (p < 0.001, t-test, Supplementary Fig. 1B,C). These two components accounted for 74% of the variance in the data so were the only ones considered. This second component is highly correlated with the template of noxious-evoked brain activity (r^2^ = 0.97, Supplementary Fig. 1B), demonstrating that the template of noxious-evoked brain activity^6^, projected onto the time window 200–500 ms after the stimulus, is suitable for calculating the magnitude of noxious-evoked brain activity in response to experimental noxious stimuli on the hand in term infants.

#### Statistics

Statistical analysis was performed in MATLAB R2014b (MathWorks). Analysis of the noxious-evoked brain activity was performed on the individual subject average response, and multiple linear regression analyses were conducted with (1) mode of delivery, sex and gestational age; (2) the logarithm (base 10) of the copeptin concentration and gestational age; (3) fetal distress, time since rupture of membranes, length of second stage of labor, maternal regional anesthesia during labor, sex and gestational age (within the vaginal group), included as independent variables. Normality of the residuals was confirmed using Q-Q plots. Comparison of pH between the two groups was carried out using Mann-Whitney-U tests, and the logarithm base 10 of the copeptin concentration was compared using a t-test.

## Supplementary information

Supplementary Material
