# Supplementary material for: Birth experience in newborn infants is associated with changes in nociceptive sensitivity
Source: Sci Rep. 2019 Mar 11;9:4117. doi: 10.1038/s41598-019-40650-2 (PMC6412011; doi:10.1038/s41598-019-40650-2)
Supplement: Supplementary file 1 — Supplementary Material [file 41598_2019_40650_MOESM1_ESM.pdf]

# Birth experience in newborn infants is associated with changes in nociceptive sensitivity – Supplementary Material

Severin Kasser, Caroline Hartley, Noemi Klarer, Hanna Rickenbacher, Antoinette Depoorter, Alexandre N Datta, Maria M Cobo, Sezgi Goksan, Amy Hoskin, Walter Magerl, Evelyn A Huhn, Gabrielle Green, Rebeccah Slater, Sven Wellmann

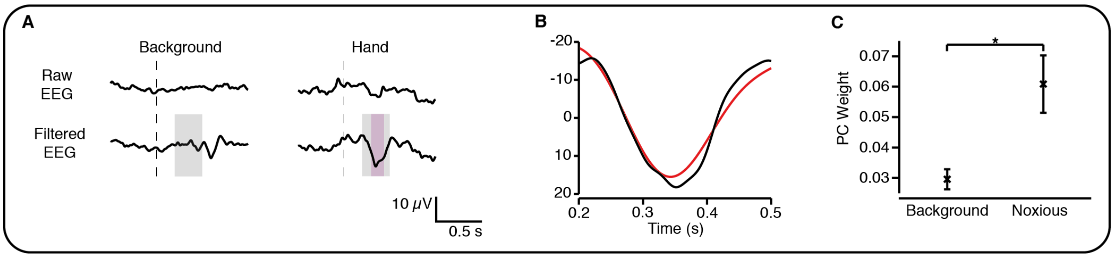


**Supplementary Figure 1: Identifying the latency of the noxious-evoked brain activity in response to stimulation on the hand.**
(A) Raw average EEG across all infants and the (Woody) filtered EEG in response to experimental noxious stimuli applied to the hand, and in background brain activity. The data was Woody filtered to the group average in the region 0 – 0.7 seconds. Black dashed lines indicate the point of stimulation. Pink shaded area indicates the significant cluster of activity identified using non-parametric cluster analysis comparing the stimulus response with background activity. Grey shaded region (which encompasses the pink region) indicates the waveform of the entire evoked response following stimulation on the hand and the same time window in the background activity. Principal Component Analysis was then conducted in this time window to identify characteristic waveforms. (B) The waveform of the second principal component (black) identified using Principal Component Analysis conducted in the time window 200 – 500 ms after the stimulus in response to stimulation of the hand compared with background activity. This is highly correlated with the previously described template of noxious-evoked brain activity (red). (C) The weights of the second principal component were significantly higher following noxious stimulation compared with in the background brain activity (*: $p<0.001$).
